# Mr. Hogben, on Early Discharge of Liquor Amnii

**Published:** 1802-09-01

**Authors:** James Hogben

**Affiliations:** Berner's Street


### *To the Editors of the Medical and Phyſical Journal.*

Gentlemen,

SHOULD you think the following paper worthy of being inſerted in your extenſive and very uſeful publication, it is at your ſervice.

It contains the caſe of a lady, who being between three and four months advanced in pregnancy, although the membranes broke, and the waters diſcharged, yet ſhe went the full time of geſtation.

Mrs. P——, about forty years of age, of middle ſtature and active diſpoſition, having been ill ſeveral weeks with colds, inflamed glands about the neck and throat, cough, and much loſs of appetite, which had reduced and weakened her greatly. On the 10th day of November 1801, being alarmed in the evening by the falling of an iron bar belonging to a window cloſe under where ſhe was in bed, was ſeized with ſuch violent ſpaſmodic efforts, as to occaſion the rupture of the membranes ſurrounding the child. The next morning, being deſired to attend, I was informed there had been a diſcharge of waters, accompanied with pain. On examining, the os internum appeared cloſed, but deſcended; and the os externum ſomewhat relaxed.

November 17th. This being the eighth day, the diſcharge and pains continued, but with ſhort intermiſſion, until the laſt twenty-four hours, having been free from both during that time; ſince which, the waters have diſcharged in much ſmaller quantities, and the motion of the child is almoſt imperceptible. Since the rupture of the membranes, the diſcharge of waters was ſo copious, as for ſeveral times to run through ſixteen folds of blankets and the bed, to the floor; by which the patient became ſo much reduced in ſize, as not to appear in a ſtate of pregnancy; although, before the accident, ſhe ſeemed much larger than uſual at that ſtage of geſtation. Being importuned yeſterday, November 16th, to eat a bit of boiled haddock, it ſo diſordered her ſtomach and whole frame, as nearly to have proved fatal. This obſervation is only to ſhow, with what caution even light ſolids, and in ſmall quantities, ſhould be allowed, when there is a general debility with indiſpoſition; even ſhould there be an inclination to food without the power to digeſt it.

Nov. 20th. The eleventh day, I examined again, and perceived the os uteri to be lower down, nearly touching the os externum, externum, but not open or soft; the pains returning frequently, attended with a discharge of waters and a bearing down at times still, as if the womb was forcing from her, (as she expressed). Being now exceedingly languid, she was under the necessity of taking frequently wine and water, broth, beef tea, &c. to support nature; and yet she was with some difficulty kept from fainting.

My patient and her friends now were much alarmed, and begged of me to relieve her from her deplorable state, by assisting the delivery of the child, which they concluded was necessary to save the mother's life, and the sooner the better. My reply was, that I could not be justifyed in hastening the delivery; in so doing the child would be destroyed, and I could not answer for the mother in her present state of health. I encouraged her by every means to have patience; and I assured her all would terminate favourably in due time. Previous to her fright, she had been taking balsamic anodyne medicines, and using gargles; as the mucus discharged from the glands about the throat was uncommonly viscid, and adhered so much to the œsophagus and epiglottis, that she was under the greatest apprehensions of being suffocated. The pulse during her long indisposition, has indicated but little fever. (The opium, &c. were still continued.)

21st. The os externum was more lax; the os uteri continued closed, and not soft; the motion of the child was perceived.

22d. Had a better night; more sleep, but frequent pains; and the waters continued discharging. The child now appeared to the mother to become more languid.

23d. She had a pretty good night; less pain, and was able to sit up; her pulse is good, and her cough better.

25th. She had a tolerable night, but the pains and bearing down increased, occasioned by some surprise; she was out of bed, and rather cheerful.

27th. She had again a restless night, much pain and bearing down; so violent, that the expulsion of the fœtus was expected every moment, but no waters were discharged. The child again appeared weaker, its motion a sort of fluttering. She is much better in health, takes light nourishment, and is out of bed some hours in the day.

28th. Had a better night; more sleep, less pain; some discharge, resembling the whites.

30th. Again had a very restless night; and pains, accompanied with a discharge of waters, thicker in consistence at times. On examining, I found the os internum soft, and a little open, but not enough to introduce the point of a finger; the neck of the uterus seemed distended quite to the mouth, and its contents refting clofe over it. The os externum continues very lax.

December 1ft. She flept better; the pains are moderate.

3d. There was a more copious difcharge of waters in the night, attended with forcing pains; yet fhe flept more, but felt herfelf this morning very weak and low. She perceives the motion of the child to be ftronger, preffing upwards againft the ftomach, which in fome degree affects her refpiration, and makes her appear to herfelf as if fhe was fuller and enlarged. Eleven o'clock at night I examined again, and found the os internum much higher, clofed, not foft, and the uterus contracted. She has been out of bed more to day than ufual; the pains trifling, but the waters were conftantly draining off.

5th. The waters continued difcharging; the child was perceived ftronger, and much agitated; fometimes it feemed to her preffing againft the ftomach; at others to be forcing downwards, but the pains moderate.

6th. An exceedingly reftlefs night; difcharge of waters, with bearing down pains.

7th. No fleep all night; bearing down pains, the waters difcharging at the fame time rather thicker, and of a brown yellow colour; the child is perceived to move at times.

8th. By taking more opium laft night, fhe flept better; but the difcharge as ufual.

9th. This morning the child was perceived lower down; its motions lefs; the difcharge thicker, but not fo much of the yellow caft. Eleven o'clock at night, has been out of bed the greateft part of the day; the pains this day have been but few and moderate, without any bearing down. The child continues to be low down, and weighty; its motion but little, and the difcharge lefs; her health and fpirits, upon the whole, better.

11th. Continues much better in health; up the greateft part of the day; very little difcharge, and that not very thin. The child moved but feldom, but it then gave pain, and at times a bearing down, as if fomething had been coming away.

12th. She was able to walk about the room; felt much ftronger; the child moved but feldom. Had in the courfe of the day two or three violent pains in the back like cramp, (fuppofed to have taken cold); the difcharge not much, but thicker, and of a brown yellow caft.

13th. The violent pains in the back were this day fomewhat abated; and the child moved very gently (with a kind of fluttering motion); the difcharge yellowifh.

14th. She had a reftlefs night; fome waters difcharged with pains; the child moved ftronger, and occafioned much pain at the the time with a bearing down. Omittèd the opiate the two preceding nights; repeated it again this morning.

15th. A much better night; was cheerful, and appeared pretty well, fitting up; fome waters pafs with a thickifh difcharge at times; fhe felt the child ftronger.

16th. A very difturbed night; pains and bearing-down; fuppofed two or three quarts of waters, with a thickifh difcharge, came off in the courfe of the night; and fince the membranes were firft ruptured, it is calculated there could not have been lefs than five or fix gallons of waters difcharged. They got vent chiefly in the night, when in an horizontal pofition: The child, coming in clofer contact with the uterus, gave greater pain by its ftruggles, and occafioned the uterus to contract, confequently difcharged the waters; or the rupture in the membranes may be high up, fo that the waters could not fo eafily get vent in a perpendicular pofition. There has not been any appearance of blood.

17th. A better night; no waters; but the thick, yellowifh difcharge in a very fmall quantity. The child was in frequent motion, but weak, and feemed to the mother low down; now and then fharp pains in the back. For fome days complained of great coldnefs in the external part of the abdomen, about the navel, which cannot be removed by hot flannels, &c. This moft likely proceeds from cold taken; which, no doubt, was the caufe of the violent cramp-like pains in the back on the 12th and 13th (the weather being intenfely cold.)

21ft. No difcharge of waters; but the yellow, thickifh difcharge continues, with pain in the back and womb frequently.

24th. The motion of the child is gentle; yet caufes pain in the womb. Having a better appetite, fhe recovered ftrength, walked about the houfe, and was cheerful; yet felt, at times, as if fhe was going to be delivered.—(Opium repeated, p. r. n.)

29th. She continued getting much better in health, and feemed ftronger than fhe had been for fome time; ate as in common with the reft of the family; flept better, and looked better in her face. The motion of the child ftill gives pain. It feems to rub or grate againft the womb (fo fhe expreffed herfelf). The yellowifh difcharge continued; fhe appeared to herfelf fometimes very big, at others quite fmall. This may be occafioned by the child being higher or lower. The cold complained of in the belly has left her.

31ft. In good fpirits; but in confequence of a fire a few doors off, the preceding day, fhe had an hyfteric fit; yet no ill effects followed. The difcharge as ufual, thickifh and yellow; frequently felt the child, but only in a kind of fluttering motion, very different (ſhe obſerved) from what ſhe uſed to feel in her former pregnancies, when the waters had not been diſcharged.

1802.—Jan. 12th. Continues in pretty good health, excepting ſlight colds affecting the head, face, and throat; her appetite was better than it had been for ſome time. The yellowiſh diſcharge as uſual; the child's motion weak, giving but little pain.

18th. The child is ſtronger, and gives more pain; the diſcharge as before, excepting ſome few red ſpots, which ſhe obſerved this morning. She appeared again much bigger, and felt ſo to herſelf.

Feb. 6th. In good health; walked about in the air, and rode in a coach, but complained of much pain when the child moved: The diſcharge now is much thicker, but yellowiſh.

25th. Her health good; the child ſeemed to pant, ſtruggle, and gave much pain when ſhe walked or ſtood, ſo as to be fatigued.

March 6th. Had a fall the other day, ſince which there has been conſiderable diſcharge of waters, and the motion of the child has been more painful.

16th. An alarm yeſterday from the cry of fire, occaſioned ſickneſs, tremblings, and great pain many hours, as if in labour, with conſiderable watery diſcharge, and the child ſeemed to her to pitch very low.

18th. Tolerably eaſy; felt the child move again, and raiſed up, preſſing againſt the ſtomach.

25th. For ſeveral nights paſt pains came on like labour, about one o'clock, and continued the greateſt part of the night, attended with the yellow watery diſcharge. She has taken cold, and complains of ſore throat, &c.

April 12th. Pains continue, with ſome diſcharge as uſual, The pains in the back, no doubt, are rheumatic.

19th. Frequent grinding pains in the back.

20th. Taken laſt night, about eleven o'clock, with ſharp pains, which have continued more regular, and attended this morning with a diſcharge of mucus. Theſe pains increaſed till between ſeven and eight o'clock in the evening; at which time ſhe was delivered of a ſtrong, healthy, full-grown male child; the head preſented; the membranes preceded, and protruded quite to the os externum, when they ruptured with a pain; about the quantity of a tea cup full of water diſcharged; the head immediately followed, and with a little aſſiſtance the child was delivered.

21ſt. I was informed by the nurſe there appeared to be about the uſual quantity of diſcharge when put to bed, as in her former labours, labours. On examining the placenta and membranes, I could not discover any other aperture in the membranes but that through which the child passed, in the most depending part, directly opposite to the placenta. In no one labour before had she so good a time in getting about; she was able to sit up the second day; and at the end of the tenth day walked about the room. She suckles the child, and has great plenty of milk.

I believe it is not common, at so early a state of gestation, as between the third and fourth months, for pregnancy to proceed long after a rupture of the membranes. In this case there being an unusual quantity of the waters so early, there can be no doubt they must have accumulated from time to time. It seems also certain, that the adhesion of the placenta to the uterus must have been uncommonly firm, and the make and texture of the child singularly compact; but that it should have survived the almost constantly repeated contraction of the uterus for so long a time, one cannot easily comprehend. The yellowish discharge might be occasioned by the contents of the child's bowels being pressed out from time to time by the violent contraction of the uterus, and so tinging the waters, &c. I examined the state of the uterus, &c. very seldom, and then as gently as possible, well knowing the less this is done under such circumstances the better: Indeed, it is seldom any manual assistance can be given early in pregnancy, even in cases attended with floodings. Nature alone generally expels the contents of the uterus in abortions. The opium administered in small quantities, and frequent, no doubt, assisted greatly in quieting the pains and action of the uterus. Indeed, without it I do not conceive the life of the child could have been preserved. It may not be improper to remark, that the patient increased in size latterly, so as to be, at the end of the ninth month, about the same bulk as in her former pregnancies. She always quickened very early, (i. e.) about the beginning of the third month. The child is now in good health, and thrives much

I am, &c.

*Berner's Street, Aug.* 18, 1802. JAMES HOGBEN.

